# The Fruit Intake–Adiposity Paradox: Findings from a Peruvian Cross-Sectional Study

**DOI:** 10.3390/nu15051183

**Published:** 2023-02-27

**Authors:** Jamee Guerra Valencia, Willy Ramos, Liliana Cruz-Ausejo, Jenny Raquel Torres-Malca, Joan A. Loayza-Castro, Gianella Zulema Zeñas-Trujillo, Norka Rocío Guillen Ponce, Fiorella E. Zuzunaga-Montoya, Mario J. Valladares-Garrido, Víctor Juan Vera-Ponce, Jhony A. De La Cruz-Vargas

**Affiliations:** 1 Instituto de Investigaciones en Ciencias Biomédicas (INICIB), Universidad Ricardo Palma, Lima 15039, Peru; 2 Facultad de Ciencias de la Salud, Universidad Privada del Norte, Lima 15314, Peru; 3 Instituto de Investigaciones Clínicas, Universidad Nacional Mayor de San Marcos, Lima 15001, Peru; 4 Facultad de Ciencias de la Salud, Universidad Científica del Sur, Lima 15067, Peru; 5 South American Center for Education and Research in Public Health, Universidad Norbert Wiener, Lima 15046, Peru; 6 Facultad de Psicología, Universidad Tecnológica del Perú, Lima 15046, Peru

**Keywords:** obesity, fruit, waist circumference, body mass index

## Abstract

Due to the increase in obesity worldwide, international organizations have promoted the adoption of a healthy lifestyle, as part of which fruit consumption stands out. However, there are controversies regarding the role of fruit consumption in mitigating this disease. The objective of the present study was to analyze the association between fruit intake and body mass index (BMI) and waist circumference (WC) in a representative sample of Peruvians. This is an analytical cross-sectional study. Secondary data analysis was conducted using information from the Demographic and Health Survey of Peru (2019–2021). The outcome variables were BMI and WC. The exploratory variable was fruit intake, which was expressed in three different presentations: portion, salad, and juice. A generalized linear model of the Gaussian family and identity link function were performed to obtain the crude and adjusted beta coefficients. A total of 98,741 subjects were included in the study. Females comprised 54.4% of the sample. In the multivariate analysis, for each serving of fruit intake, the BMI decreased by 0.15 kg/m^2^ (β = −0.15; 95% CI −0.24 to −0.07), while the WC was reduced by 0.40 cm (β = −0.40; 95% CI −0.52 to −0.27). A negative association between fruit salad intake and WC was found (β = −0.28; 95% CI −0.56 to −0.01). No statistically significant association between fruit salad intake and BMI was found. In the case of fruit juice, for each glass of juice consumed, the BMI increased by 0.27 kg/m^2^ (β = 0.27; 95% CI 0.14 to 0.40), while the WC increased by 0.40 cm (β = 0.40; 95% CI 0.20 to 0.60). Fruit intake per serving is negatively related to general body adiposity and central fat distribution, while fruit salad intake is negatively related to central distribution adiposity. However, the consumption of fruit in the form of juices is positively associated with a significant increase in BMI and WC.

## 1. Introduction

Obesity and its associated comorbidities have shown a marked global increase between 1980 and 2008 in some regions, such as Latin America [1]. In this context, international organizations such as the World Health Organization (WHO) have begun to promote the adoption of a healthy lifestyle, as part of which daily fruit and vegetable intake of five or more servings per day stands out for the prevention of non-communicable diseases [2]. Culinary and nutritional aspects of fruit consumption may differ in comparison to vegetable consumption [3,4]. For instance, fruits are usually consumed raw, as desserts, in juices, or in other presentations which potentially favor their intake relative to that of vegetables. In addition, the nutritional composition of fruits and vegetables differs since their fructose and water content can vary [3,4].Despite evidence points out a negative association between fruit intake and different health outcomes such as type 2 diabetes mellitus (DM2), hypertension, cancer and depression [5,6,7,8], fruit consumption has shown a downward trend in countries of the Latin American region [9]. In Peru, a country with high variability in dietary patterns among different areas, it has been reported that fruit and vegetable intake is insufficient to the extent that only 4.8 to 11.2% of the population over 18 years old reaches the minimum recommendation of 5 servings per day [9,10].

Postulated mechanisms by which fruit intake exerts a regulatory role in body weight management include displacement of energy-dense foods; reduction in total energy intake; increase in satiety and satiation; and higher contribution of fiber, micronutrients and phytochemicals to the diet [3,11,12]. However, research analyzing the relationship between weight loss or obesity prevention and fruit consumption has shown great variability. For example, a systematic review that included randomized controlled trials reported that, in most of the studies, an increase in fruit intake, but not that of vegetables or that of fruits and vegetables, reduces the body weight, and/or waist circumference (WC) [3]. Furthermore, prospective and cross-sectional observational studies report an inverse association with weight gain or development of overweight-obesity for the consumption of fruits but not for the consumption of vegetables or fruits and vegetables together [3,13,14]. It should also be considered that the measurement of obesity is highly variable, as it can be assessed through different anthropometric markers such as the body mass index (BMI) and/or the WC [15].

To the best of our knowledge, few studies have analyzed the relationship between fruit intake and anthropometric markers of adiposity in the Latin American region. Furthermore, it has been reported that general obesity and central obesity prevalence are stepping increasing worldwide and particularly the South American countries are the most affected ones [16,17]. In addition, in the existing studies, methodological approaches such as representing the consumption of fruits and vegetables as a dichotomous variable, have failed to yield a significant association [18]. Since there is evidence of a dose-response relationship between fruit consumption and other health outcomes [6,7,8,9,19,20] and there is a knowledge gap on the topic, the current study aims to analyze the association between fruit intake and body mass index and waist circumference in a representative sample of Peruvians.

## 2. Methods

### 2.1. Study Design

This was a cross-sectional analytical study. Secondary data analysis was conducted using information from the Demographic and Health Survey of Peru (ENDES). For this manuscript, the data collected in the years 2019, 2020 and 2021 were analyzed. The STROBE (Strengthening the Reporting of Observational studies in Epidemiology) guidelines were followed for the present study [21].

### 2.2. Population and Sample

The ENDES is a nationally representative survey with a two-stage sampling design (National Institute of Statistics and Informatics, 2015). The sample was characterized as being probabilistic of a balanced, stratified, and independent type, at the departmental level and by urban and rural area.

### 2.3. Definition of Variables

The outcome variables were BMI and WC. BMI was calculated using the weight × height^2^ formula. Height was measured with a mobile, multipurpose wooden stadiometer with a precision of 1 mm and with the technical specifications of the National Food and Nutrition Center (CENAN by its acronym in Spanish). A SECA-878 brand scale was used to measure body weight with a precision of 50 g. For the waist circumference, a Lufkin brand retractable metal tape with a resolution of 0.1 cm was used. In addition, the anthropometric techniques recommended by the WHO were used to measure weight, height, and waist circumference. The latter was measured as the mean distance between the last costal margin and the upper edge of the iliac crest, as previously reported in the ENDES anthropometrist manuals [22].

The exploratory variable was fruit intake, which was self-reported and expressed in number of servings per day in three different presentations. The first form of intake was evaluated through the question: How many units, slices, or fruit bunches did you eat per day? The consumption of fruits as a garnish was included, and, for the case of dried fruits, only raisins were considered. The second form of intake was the consumption of fruit juice per day, which was assessed through the question: How many glasses of fruit juice did you drink per day? In this case, both fruit juices and their extracts were included. Finally, the consumption of fruit in salad per day was assessed through the question: How many servings of fruit salad did you eat per day? To assess the three forms of presentation, a laminar was used as graphic support [23] with portion sizes and standardized household measurements. The technical aspects of collecting this information have been previously published in manuals by the National Institute of Statistics and Informatics (INEI by its acronym in Spanish) [24].

The factors to be evaluated were sex (male vs. female); categorized age (15–34, 35–60, 61–69, and ≥70 years); educational level (no level, primary, secondary, and higher); wealth index (poorest, poor, medium, rich and richest); natural region (Metropolitan Lima, rest of the coast, mountains and jungle); daily tobacco use (yes vs. no); self-reported alcohol consumption in the previous 12 months (yes vs. no); history of type 2 diabetes mellitus (yes vs. no) and arterial hypertension (yes vs. no).

### 2.4. Statistical Analysis

The statistical software SPSS 26 was used. The median and interquartile range of each form of presentation of fruit intake were estimated. BMI and WC were presented with their respective 95% confidence intervals (95%CI). The descriptive variables were presented in absolute and relative frequencies. Student’s *t* test/one way ANOVA was used for the bivariate analysis, and Spearman’s correlation was used for the main variables. Finally, a generalized linear model of the Gaussian family and identity link function were performed to obtain the crude and adjusted beta coefficients. All the analyses were carried out considering that the samples were complex.

### 2.5. Ethical Aspect

This study was developed by analyzing survey data sets that are openly published and available online with all identifier information removed (http://iinei.inei.gob.pe/microdatos/, accessed on 16 November 2022). Furthermore, all survey data were coded to ensure anonymity to minimize potential harm.

## 3. Results

A total of 98,741 subjects were included in the study. The Females comprised 54.4% of the study population. A total of 33.6% of the subjects lived in Metropolitan Lima. A total of 20.2% were over 60 years of age. The mean BMI was 27.3 ± 4.9 kg/m^2^, while for the WC, it was 58.7 ± 8.2 cm. Regarding fruit intake, the mean portions of whole fruit, juices, and salad, was 1.4 ± 1.3; 1.5 ± 0.8 and 1.2 ± 0.6, respectively. Table 1 shows the whole study sample characteristics, disaggregated by sex. Differences in BMI, WC, history of hypertension and DM2 were statistically significant between sexes.

In the bivariate analysis, a statistically significant association was found between each characteristic and BMI and WC (Table 2).

In the multivariate analysis (Table 3), for each serving unit of fruit intake, the BMI decreased by 0.15 kg/m^2^ (β= −0.15; 95% CI −0.24 to −0.07), while the WC was reduced by 0.40 cm (β = −0.40; 95% CI −0.52 to −0.27). In the case of fruit juice, for each glass of juice consumed, the BMI increased by 0.27 kg/m^2^ (β = 0.27; 95% CI 0.14 to 0.40), while the WC increased by 0.40 cm (β = 0.40; 95% CI 0.20 to 0.60). Finally, a negative relation between fruit salad intake and WC was found, with a 0.28 cm decrease for each serving (β = −0.28; 95% CI −0.56 to −0.01). However, this was not the case for BMI (β= −0.10; 95% CI −0.28 to 0.08).

Intake of three or more fruit portions was associated with a significant decrease in BMI and WC when compared to less than 3 portions intake (Table 4). A decline of 0.24 kg/m^2^ (β= −0.24; 95% CI −0.32 to −0.17) and 0.60 cm (β = −0.60; 95% CI −0.72 to −0.47), was found for BMI and WC, respectively

## 4. Discussion

### 4.1. Main Findings

The present study found evidence of a relationship between fruit intake and anthropometric indicators of adiposity. However, the magnitude and direction of the relationship varied depending on the presentation of fruit consumption. Consumption of each portion of fruit resulted in a negative beta coefficient for BMI and waist circumference. A negative relation for fruit salad intake and WC was found; in contrast, the consumption of fruit juices and extracts was related to an increase in these indicators.

### 4.2. Comparison with Other Studies

Fruit intake in portions per day was lower than that reported by previous studies from Canada and European countries, with the average intake found to be between 1.8 and 2.4 portions per day [25,26]. In contrast, our findings are consistent with the ELANS study (Latin American Study of Nutrition and Health), in which the intake of fresh fruit in Latin American countries varied from less than one serving in Venezuela to 1.5 servings per day in Peru [9]. Our findings regarding fruit juice intake were similar to those of the ELANS study, although in that research, fruit juices and other homemade beverages were considered in the same category [9]. The comparison of fruit juice intake between studies is more complex due to the variable methods of reporting consumption (servings per day with different household measurements as standard measurements vs. grams per day). It is particularly noteworthy that fruit intake in Peru occurs not only in the form of whole fruits; rather, the intake of fruit salad is also a popular alternative, which is why there are businesses that are in charge of selling fruit and fruit salads. However, as there are no reports of consumption of this fruit presentation at the national level, it was not possible to compare the intake of fruits in this culinary presentation form with other studies.

Consuming more servings of fruit per day was inversely related to BMI and waist circumference. These findings are in line with previous cross-sectional [27,28] and prospective [3,13,27,28,29] studies. Although differences in the magnitude of the beta coefficients between fruit intake and anthropometric markers among studies exists, it should be considered that this variability may reflect the diverse nature of the population studied. For instance, while the PREDIMED-plus cohort study (PREDIMED: Prevención con Dieta Mediterránea) [26] exclusively analyzed patients with metabolic syndrome and reported a greater beta coefficient than that reported in the present study, the study by Yu et al. [25], included healthy individuals and those with chronic diseases as the present study did. After the corresponding adjustment for diseases was made, Yu et al. found beta coefficients similar to what we found. This suggests that the modulation that fruit intake has on adiposity could be greater in people with chronic diseases, compared to those who are healthy [4].

Since a specific fruit intake dose for excess adiposity accumulation protection is not currently suggested by any international organization or dietary guidelines, we decided further to analyze the relationship between a three-portion daily fruit intake against BMI and WC. This decision was made on the rational basis of existing evidence pointing out a protective effect of this dose against other cardiometabolic diseases [7,8]. Not surprisingly the three-portion daily fruit intake was significantly and negative associated with BMI and WC. Furthermore, the magnitude of WC reduction, this is −0.6 cm, may be considered as clinically relevant as a study revealed that each 1cm-increase of WC is associated with a 2% increased risk of cardiovascular disease (CVD) [30]. Inference from that study could be made in a reverse way, such that the observed current reduction of 0.6 cm (95% CI −0.72 to −0.47) could be associated with a CVD risk reduction of 1% (ranging 0.9–1.5%). In this sense, our findings confirm the benefits that intake of three or more fruit portions per day have over cardiometabolic health and add evidence to previously published studies regarding the dose-dependent protective effects of fruit intake on DM2 and cardiovascular diseases prevention [7,8].

Fruit salad consumption was also negatively correlated with waist circumference but not with BMI. The reason for the lack of statistically significant correlation with BMI may lie in the fact that adding sugar-sweetened yogurts and energy-dense accompaniments is a frequent practice in the commercial preparation of fruit salads in Peru. Therefore, an increase in total energy intake may have neglected statistically significant association when analyzing total body adiposity with BMI. On the contrary, the potential presence of added sugar in fruit salad may have been overpowered by the fiber content when central adiposity was analyzed through waist circumference. However, as the presence of added sugar components was not assessed, this cannot be confirmed.

In contrast to what was found for fruit consumption per serving, the intake of fruit juices per day was positively related to an increase 0.27 kg/m^2^ and 0.40 cm of BMI and waist circumference, respectively, for each serving of juice consumed. This finding differs from that found in other works in which the intake of natural fruit juices showed an inverse and dose-dependent relationship with BMI and waist circumference [25,26,27,28,29,30,31,32,33]. Methodological differences in the assessment of fruit juice intake may partly explain this discrepancy. While some studies differentiated the consumption of natural fruit juice from packaged fruit juice [26,32,33], and others included both fruit and vegetable juices in the category of juices [24], the present work included both juices and fruit extracts, the latter being low in fiber content and more concentrated in sugars. Additionally, a core aspect to consider is that adding refined sugar is part of traditional fruit juice preparation in Peru, as well as in other Latin American countries [9], which increases caloric intake and reduces the protective effect against the development of adiposity. In line with the above, a systematic review analyzed the risk of developing type 2 diabetes mellitus for the consumption of fruit juices with and without added sugar and reported a higher risk for the former, but not for the latter [34].

The present study found that fruit intake, but not fruit juice consumption, is inversely related to adiposity. However, it is important to consider that this effect is part of a set of dietary patterns that include the intake of less energy-dense foods that are richer in micronutrients, phytochemicals, and fiber. In support of this, a prospective study with 5.5 years of follow-up found that the change in “waist circumference for a given BMI” was −0.04 cm/year for fruit intake, but the magnitude of the change rose to −1.1 cm/year when all other food groups that prevent the gain of central adiposity were included, which are characteristically higher in fiber and micronutrients and less energy-dense [29]. In this way, the evidence suggests that the mechanisms by which fruit consumption reduces body adiposity, particularly that of central distribution, are related to the more significant contribution of fiber and micronutrients that modulate satiety and intestinal microbiota and displace the consumption of foods with a high content of saturated fats, sugars and sodium [26,35]. 

Our research highlights the urgent need to strengthen public policies regarding fruit intake in low- to middle-income countries such as Peru in which fruit consumption, regardless of the culinary presentation, is well below what is needed for the population to benefit from its protective effect against cardiometabolic diseases. Furthermore, considering the high healthcare cost attributed to general and central obesity [36,37], its steepening trend [16,17], and the potential cost savings on its reduction [38,39], strengthening cost-effective public policies such as nutritional education targeted at vulnerable populations should be considered [40], especially when significant CVD risk reduction may be achieved with this kind of approaches. 

### 4.3. Study Limitations

This study includes limitations that must be considered. First, the study’s cross-sectional nature prevents the establishment of a causal relationship for the outcomes obtained. Second, although the analysis was adjusted for different covariates that have been reported to be important for fruit intake [41], we could not adjust for total energy intake or physical activity, covariates that are significant in analyses of food consumption [27], as the information coming from ENDES does not assess caloric intake. Third, intake was assessed via self-report and was not carried out with the most frequently used methodology, that is, with the use of the Food Frequency Questionnaire (FFQ), which may limit the possibility for comparisons between studies. However, the intake assessment was carried out with open questions about the frequency per week and portions per day of consumption of fruits, fruit salads, and fruit juices. This was based on the proposal of the World Health Organization, the STEPwise approach to noncommunicable disease risk-factor surveillance [42].

Some strengths deserve to be highlighted. The study was developed with data from a national survey and with a sample size that guarantees representativeness and gives statistical power to the reported findings. Additionally, the analysis that was carried out differentiated the consumption of fruits in their whole form, in salad presentation, and in the form of juices. This is a relevant aspect since the forms of presentation of fruit consumption analyzed in the study are part of the food culture of Peru; the study allows for the differentiation of the possible effects of each of these culinary presentations.

## 5. Conclusions

Fruit intake per serving is negatively related to general body adiposity and central fat distribution, while fruit salad intake is negatively related to central distribution adiposity. However, fruit consumption of fruit in the form of juices is positively associated with a significant increase in BMI and WC. Due to the potential displacement mechanisms of more energy-dense foods and the increased satiety generated by fruits, it is recommended that future studies assess both energy intake and expenditure. Finally, since a dose-response analysis for each intake level of fruit and obesity protection is still lacking, it is suggested that future studies be carried out in this field.

## Figures and Tables

**Table 1:** Descriptive characteristics of the total study sample by sex.

Characteristics	n (%Weighted)		
	Total 98,471 (100.0)	Masculine 43,113 (45.6)	Feminine 55,358 (54.4)
**Categorized Age**			
15 to 35 years old	45,636 (38.6)	18,349 (38.6)	27,287 (38.7)
36 to 59 years old	38,381 (41.1)	18,004 (41.3)	20,377 (41.0)
60 to 69 years old	8015 (11.3)	3707 (11.1)	4308 (11.6)
70 years old or more	6439 (8.9)	3053 (9.0)	3386 (8.8)
**Natural region**			
Metropolitan Lima	39,153 (33.6)	5108 (34.0)	6283 (33.3)
rest of coast	27,256 (25.2)	12,044 (25.5)	15,513 (24.9)
mountains	25,950 (27.6)	15,475 (26.5)	20,645 (28.5)
Jungle	13,152 (13.6)	10,486 (14.0)	12,917 (13.2)
**Education Level**			
No level	179 (0.2)	50 (0.1)	129 (0.3)
Primary	22,556 (20.9)	9269 (18.5)	13,287 (22.8)
Secondary	43,953 (42.7)	20,651 (46.4)	23,302 (39.7)
Higher No specified	27,716 (32.3) 4067 (3.9)	12,522 (33.8) 621 (1.2)	15,194 (31.1) 3446 (6.1)
**Wealth index**			
The poorest	31,899 (21.7)	14,306 (22.1)	17,593 (21.4)
Poor	24,790 (21.2)	10,830 (21.7)	13,960 (20.8)
Medium	18,011 (20.0)	7675 (19.6)	10 336 (20.3)
Rich	13,690 (18.7)	5839 (18.0)	7851 (19.3)
Richest	10,081 (18.4)	4463 (18.6)	5618 (18.2)
**Smoke daily**			
Yes No	1090 (1.4) 97,381 (98.6)	882 (2.4) 42,231 (97.6)	208 (0.5) 55,150 (99.5)
**Alcohol consumption**			
Yes No	8071 (10.2) 90,358 (89.8)	5992 (15.8) 37,121 (84.2)	2079 (5.5) 53,279 (95.5)
**History of hypertension**			
Yes No No specified	8142 (10.9) 90,231 (89.0) 98 (0.1)	3067 (9.1) 40,011 (90.8) 35 (0.1)	5075 (12.5) 50,220 (87.4) 63 (0.1)
**History of DM2**			
Yes No No specified	3223 (4.8) 95,140 (95.1) 108 (0.1)	1329 (4.4) 41,743 (95.5) 35 (0.1)	1894 (5.1) 53,397 (94.8) 67 (0.1)
Body Mass Index (kg/m^2^) *	27.3 (4.9)	26.6 (4.5)	27.7 (5.1)
Waist circumference (cm) *	58.7 (8.2)	56.5 (7.3)	60.6 (8.4)
Fruit intake (portion) *	1.4 (1.3)	1.5 (1.4)	1.4 (1.3)
Fruit intake (juices) *	1.5 (0.8)	1.6 (0.8)	1.5 (0.7)
Fruit intake (salad) *	1.2 (0.6)	1.2 (0.6)	1.2 (0.5)

* Mean and standard deviation.

**Table 2:** Bivariate characteristics of the factors associated with BMI and WC.

	Body Mass Index	*p*	Waist Circumference	*p*
	Mean ± SD		Mean ± SD	
Sex *				
Masculine	26.7 ± 4.5	<0.001	56.5 ± 7.3	<0.001
Feminine	27.7 ± 5.1		60.6 ± 8.4	
Categorized age **				
15 to 35 years old	26.0 ± 4.8	<0.001	54.7 (7.5)	<0.001
35 to 60 years old	28.5 ± 4.8		60.8 (7.3)	
60 to 69 years old	27.9 ± 4.8		62.4 (7.8)	
70 years old or more	26.5 ± 4.6		61.8 (7.9)	
Natural region **				
Metropolitan Lima	27.8 ± 5.1	<0.001	59.1 ± 8.3	<0.001
rest of coast	28.0 ± 5.0		59.7 ± 8.2	
Mountains	26.2 ± 4.4		57.7 ± 7.8	
Jungle	26,6 ± 4.7		57.6 ± 7.8	
Education Level **				
No level	26.4 ± 4.3	<0.001	61.4 ± 7.9	<0.001
Primary	27.3 ± 4.8		61.0 ± 8.1	
Secondary	27.2 ± 5.0		57.9 ± 8.4	
Higher	27.5 ± 4.8		58.6 ± 8.1	
Wealth index **				
the poorest	25.6 ± 4.3	<0.001	57.1 ± 7.7	<0.001
Poor	27.2 ± 4.8		58.8 ± 8.2	
Medium	27.9 ± 5.1		59.5 ± 8.4	
Rich	28.0 ± 5.0		59.5 ± 8.2	
Richest	27.7 ± 5.0		58.7 ± 8.2	
Smoke daily *				
Yes	27.3 ± 5.1	<0.001	57.2 ± 7.9	<0.001
No	27.2 ± 4.9		58.7 ± 8.2	
Alcohol consumption *				
Yes	27.6 ± 4.9	<0.001	57.5 ± 7.5	<0.001
No	27.2 ± 4.9		58.8 ± 8.2	
History of hypertension *				
Yes	29.3 ± 5.7	<0.001	64.0 ± 8.0	<0.001
No	27.0 ± 4.8		58.1 ± 7.9	
History of DM2 *				
Yes	29.1 ± 5.1	<0.001	63.6 ± 7.8	<0.001
No	27.2 ± 4.7		58.5 ± 8.1	

* Analysis performed with Student’s *t* test. ** Analysis performed with one-way ANOVA.

**Table 3:** Simple and adjusted multivariate regression analysis of the association of fruit consumption with BMI and WC.

Characteristics	Crude Analysis			Adjusted Analysis *		
	Coeff. Beta Crude	CI 95%	*p*	Coeff. Beta Adjusted	CI 95%	*p*
**Body Mass Index**						
Fruit intake (portion)	−0.14	−0.17 to −0.11	<0.001	−0.15	−0.24 to −0.07	**<0.001**
Fruit intake (juices)	0.10	0.02 to 0.16	<0.001	0.27	0.14 to 0.40	**<0.001**
Fruit intake (salad)	−0.17	−0.32 to 0.03	<0.001	−0.10	−0.28 to 0.08	0.257
**Waist circumference**						
Fruit intake (portion)	−0.50	−0.55 to −0.46	<0.001	−0.40	−0.52 to −0.27	**<0.001**
Fruit intake (juices)	−0,23	−0.34 to −0.12	<0.001	0.40	0.20 to 0.60	**<0.001**
Fruit intake (salad)	0.39	−0.63 to −0.14	0.002	−0.28	−0.56 to −0.01	**0.045**

* Adjusted for sex, categorized age, natural region, educational level, wealth index, daily smoking, alcohol consumption, history of hypertension, history of DM2. 95% CI: 95% confidence interval.

**Table 4:** Three or more fruit portion intake multivariate regression analysis for BMI and WC with different cofounders.

Characteristics	Crude Analysis			Adjusted Analysis *		
	Coeff. Beta Crude	CI 95%	*p*	Coeff. Beta Adjusted	CI 95%	*p*
**Body Mass Index**						
Fruit intake (≥3 portions)	−0.14	−0.17 to −0.11	<0.001	−0.24	−0.32 to −0.17	**<0.001**
Fruit intake (<3 portions)	Ref					
**Waist circumference**						
Fruit intake (≥3 portions)	−0.50	−0.55 to −0.46	<0.001	−0.60	−0.72 to −0.47	**<0.001**
Fruit intake (<3 portions)						

* Adjusted for sex, categorized age, natural region, educational level, wealth index, daily smoking, alcohol consumption, history of hypertension, history of DM2. 95% CI: 95% confidence interval.

## Data Availability

Publicly available datasets were analyzed in this study. This data can be found here: https://data.mendeley.com/datasets/pkycshctcf/1.
